# The Potential of Antibiotics and Nanomaterial Combinations as Therapeutic Strategies in the Management of Multidrug-Resistant Infections: A Review

**DOI:** 10.3390/ijms232315038

**Published:** 2022-11-30

**Authors:** Oluwaseun Ola Adeniji, Nolonwabo Nontongana, Janet Chiyem Okoh, Anthony Ifeanyi Okoh

**Affiliations:** 1 SAMRC Microbial Water Quality Monitoring Centre, University of Fort Hare, Alice 5700, South Africa; 2 Applied and Environmental Microbiology Research Group (AEMREG), Department of Biochemistry and Microbiology, University of Fort Hare, Alice 5700, South Africa; 3 Department of Medicine, Faculty of Health Sciences, University of Cape Town, Cape Town 7700, South Africa; 4 Department of Environmental Health Sciences, College of Health Sciences, University of Sharjah, Sharjah P.O. Box 26666, United Arab Emirates

**Keywords:** multidrug resistance, nanoparticles, antibiotic combinations, infectious disease

## Abstract

Antibiotic resistance has become a major public health concern around the world. This is exacerbated by the non-discovery of novel drugs, the development of resistance mechanisms in most of the clinical isolates of bacteria, as well as recurring infections, hindering disease treatment efficacy. In vitro data has shown that antibiotic combinations can be effective when microorganisms are resistant to individual drugs. Recently, advances in the direction of combination therapy for the treatment of multidrug-resistant (MDR) bacterial infections have embraced antibiotic combinations and the use of nanoparticles conjugated with antibiotics. Nanoparticles (NPs) can penetrate the cellular membrane of disease-causing organisms and obstruct essential molecular pathways, showing unique antibacterial mechanisms. Combined with the optimal drugs, NPs have established synergy and may assist in regulating the general threat of emergent bacterial resistance. This review comprises a general overview of antibiotic combinations strategies for the treatment of microbial infections. The potential of antibiotic combinations with NPs as new entrants in the antimicrobial therapy domain is discussed.

## 1. Introduction

Globally, the emergence of multidrug-resistant organisms (MDROs), which account for approximately 16% of hospital-acquired infections, has been bolstered by the indiscriminate use of drugs, self-medication, and exposure to infectious diseases [1,2,3]. It is becoming increasingly difficult for physicians to care for patients with pan-drug-resistant (PDR), extensively resistant (XDR), or MDR organisms [4]. Resistance against more than one class of antibiotics characterizes the MDR organisms, whereas PDR counterparts are resistant against entirely clinical practice-recognized antimicrobial agents, established on sensitivity tests in the laboratory [5]. Patients infected with antimicrobial-resistant bacteria (ARB) require long-term antimicrobial therapy, and the prospect of effective clinical treatment deteriorates [6,7]. Hwang et al. [8] in their study described a woman who developed an actinomycotic brain abscess 15 months after being treated for non-invasive nasopharyngeal actinomycosis, which recurred as an invasive form. Carbapenems have been considered to be the most effective broad-spectrum beta-lactam antibiotic treatment against MDR Gram-negative microorganisms [4]. Consequently, colistin and tigecycline are currently regarded as “last resort” drugs for the treatment of carbapenem-resistant microorganisms. Nevertheless, as the use of these two medications has increased, there have been more reports of tigecycline- or colistin-resistant organisms emerging in the last five years [4,9,10].

The pharmaceutical manufacturing industry’s lack of investment in drug discovery because of the intrinsically low rate of profit for antibiotics versus medicines geared toward chronic illnesses exacerbated the problem challenge of increasing bacterial resistance [11]. This condition is so threatening that the World Health Organization has identified MDROs as one of the three most serious threats to human health [12,13], even though the Infectious Disease Society of America (IDSA) has issued a call for biomedical community action to address the risks associated with MDR pathogens [14]. Aside from the development of sophisticated strategies to fight MDR disease-causing organisms, several medical and paramedical disciplines will be negatively influenced [15]. As a result, other methods of regulating bacterial infectious diseases, particularly Gram-negative bacteria, are desperately needed. Also, it is imperative to develop scientific knowledge concerning antibacterial combinations and pharmacological treatments to safeguard human well-being; since the previous resolution to the spread of infections found in antibiotics is currently unexpectedly the major basis of antibiotic resistance [16]. Therefore, the primarily newly established therapies are dual antibacterial combinations with nanoparticles (NPs) [16,17]. This review aims to outline the effect of antibiotics and antibiotic combinations, the use of nanomaterials as alternate antibacterial agents in the treatment of severe bacterial infectious illnesses, nanomaterial types, synthesis methods, and the characterization, applications, and toxicity of NPs.

### 1.1. Genesis of Combination Therapy

The increase in the incidence of MDR microorganisms, especially MDR Gram-negative microorganisms, indicates that monotherapy is becoming increasingly insufficient and thus the usage of combined treatments is advocated [18]. For example, combined therapy treatments have for a long time been employed in treating patients infected with HIV [19], patients with prosthetic joint infectious disease due to *methicillin-sensitive Staphylococcus. aureus* (MSSA), and for the treatment of prosthetic heart valve infections due to coagulase-negative staphylococci [20,21]. More so, they are almost entirely used for the treatment of *Mycobacterium tuberculosis* infectious diseases and are also critical for the treatment of bacterial infections [22]. The combination of streptomycin with penicillin was reported in 1950 [23], and trimethoprim with sulfonamides in 1968 [24]. Both combinations improved efficiency and antimicrobial range. The combination of drugs to defeat the selection for resistance to single agents in the treatment of tuberculosis was documented in the early 1950s [25], whereas Noordeen [26] documented that the “advantages of antibiotic combinations for treatment of leprosy were detected in the 1960s”. Several new alternative strategies to conventional therapy regimens like bacteriophage [27], antibacterial antibodies [28], antibiotics combinations [29], photothermal treatment [30], Molecular hybridization (Synthesis of new hybrid drug) [31,32], and nanomaterials [33] have been tested.

#### 1.1.1. Antibiotic Combination Therapy

Antibiotic combination therapy is a treatment that requires the prescription and usage of multiple drugs at the same time to treat an infection [34] because the combined drugs can better defeat bacterial resistance than when the drugs are used individually [34]. The combination could work either by:

(i). Targeting diverse pathways as it has been “for the combination of isoniazid, rifampicin, ethambutol, and pyrazinamide for use in the treatment of tuberculosis” [35,36]. This combination comprises two or more drugs that individually target various facets of the infection, as many ailments have been discovered to be multicomponent or multifactorial. Also, drugs A and B’s mechanisms are very much liable to be impartial in their exact activities. Nevertheless, they could have interactive impacts on patient status’s general upturn [37].
(ii). Inhibiting different targets through a single pathway. The mechanism involves several drug combinations that aim at only one “disease component and usually a single cell type or even a single response pathway in the cell type, but with various targeted sites” [36]. This precise targeting reduced drug doses and toxicities, thus allowing more noteworthy results. For example, the drug cotrimoxazole (sulfamethoxazole + trimethoprim) utilizes two medicines functioning at various stages in a single pathway to achieve better inhibition than using either of those drugs alone [37]. “It is a combination of sulfamethoxazole, inhibits folic acid production by suppressing dihydropteroate synthetase, and trimethoprim, which functions at a later step in nucleotide production to subdue dihydrofolate reductase” [37].
(iii). Inhibiting the same target in different ways, for example, in cases regarding streptogramins and virginiamycin [35,36]. “The mode of action involves one drug that is useful on its own but is not sufficiently efficient or too toxic and a second drug that does not share the same activity as the first drug and may have no beneficial effect on its own, but that can improve the efficacy of the first drug by either pharmacokinetic/pharmacodynamic mechanism” [37].

#### 1.1.2. The Pros and Cons of Antibiotic Combination Therapy

Antibiotic combination therapy is an extremely selective procedure, although it often comprises a beta-lactam with an aminoglycoside [34]. Whether or not this establishes a positive effect for infected patients has become controversial because antibiotic combination therapy has been discovered to work in some instances. Nevertheless, the pros and cons of antibiotic–antibiotic combination therapy can help choose the best circumstances for its application [34].

##### The Pros (Benefits) of Combination Antibiotic Therapy

(a) Synergy in action: The first and most important justification for the application of combined antibiotic treatment is to generate synergistic drug interactions [37]. Synergistic combinations of antibiotics can exterminate MDR maladies much more efficiently than antibiotics taken independently [37]. For example, the tigecycline and carbapenem combination has been used to efficiently treat carbapenemase-containing *Klebsiella pneumonia*-infected patients [34]. Moreso, the combination of ceftazidime with tobramycin has been demonstrated to be a potent therapy for patients with cystic fibrosis [35,38].
(b) Polymicrobial infections: Combination therapy effectively treats polymicrobial infections—diseases that comprise multiple bacterial pathogens that are usually detected in the pelvic region, intra-abdominal and urogenital tract A diseases. Combinations of ciprofloxacin with metronidazole drugs are the basis for this treatment [39].
(c) Improved uptake and sequential blockage: In the improvement of the uptake and inhibition of consecutive steps, combining treatments also helps. Beta-lactam combined with aminoglycoside drugs results in antibacterial synergy with increased uptake. This behavior is facilitated by way of beta-lactam bringing about cell membrane impairment, which enables the aminoglycoside movement into the bacteria cells, thus improving the bactericidal activity [18].
(d) Reduced toxic effects and diminished death rate: Coherent drug combination therapy reduces the concentration necessary for therapeutics and minimizes the dose associated with toxicity and reduced mortality rate. Though, there is no information “from clinical trials that prove beyond a reasonable doubt that combination therapy with different agents allows for a reduction of the drug dose sufficient to decrease dose-related toxicity” [40].
(e) Prevention of Drug Resistance: Different molecular targets of individual agents permit the investigation of the use of drug combination therapy and thus, widen the activity spectrum. With their broad ranges of action and multimodal activity, antimicrobial substances may impede the emergence of drug resistance [18].
(f) Empirical Treatment: In a situation where the nature of the infectious disease is not evident, i.e., undiagnosed infections, empiric antibiotic combinations help begin the treatment. Utilizing empiric antibiotic therapy with an agent to which bacteria is sensitive has been linked to a decrease in death rate and improvement in the results [41,42].

##### Adverse Effects of Combination Antibiotic Therapy

Although antibiotics combination therapy has a lot of advantages, there are some significant drawbacks, which are as follows:

(i). Antagonism: In contrast to synergistic drug interactions, some combinations might show antagonism, where one drug can either invalidate or diminish the effect of the other on organisms [38]. For example, the initiation of Beta-lactamase by one agent leaves the second agent unsuccessful in treating *Enterobacter*, *Serratia*, or *Pseudomonas* with combination therapy. Antagonism may make bactericidal agent bacteriostatic [18].
(ii). *Clostridium difficile* infection (CDI): This is another known unfavorable effect of antibiotic use. Any wide-spectrum antibiotic (aminoglycosides and beta-lactams) can cause an overgrowth of *C. difficile*. “Among these, fluoroquinolones were documented being an independent risk factor for CDI’’ [43,44].
(iii). Other effects of combination therapy include: drug toxicity such as nephrotoxicity and ototoxicity, fungal overgrowth, drug interactions, irrational drug use, and a rise in the cost of treatment [42].

## 2. Drug Interactions

The drug-to-drug interactions may be pharmacokinetic or pharmacodynamic. Pharmacokinetic interactions comprise drug absorption, metabolism, distribution, and excretion, whereas pharmacodynamic interactivities describe the relationship between drug concentration on the site of action and the effect on the body. It is essential to think about various antibiotics’ reactions to discover the most effective drug combinations. The result of this combination can be “synergism (C > a + b), antagonism (C < a + b), additivity (C = a + b), autonomy (C~a or C~b), depending on whether or not the combined effect of the drugs is more than, equal to, or smaller than, the result predicted by their activities” [43,44,45,46]; where ‘a’ is drug 1, ‘b’ is drug 2, and ‘C’ is the combination of drugs 1 & 2 [36,47]. The assessment of action can be performed in-silico by the Kirby–Bauer disc diffusion method, agar-well diffusion, or checkerboard assay [36]. Synergistic combinations are more efficient, while antagonistic combinations are less effective, and additivity drug combinations are as efficient at inhibiting microbial growth as each antibiotic’s sum when used independently [48]. To quantify the interaction between the antibiotics being tested using standard checkerboard assay, “the fractional inhibitory concentration (FIC) index” value is estimated as shown below.

*“The fractional inhibitory concentration (FIC) is the Minimum Inhibitory concentration (MIC) of drug A in the company of B divided by the MIC of A”.*

In contrast, the FIC index is the sum of the FIC of drugs A and B. Often, time-kill assays are used as a follow-up to confirm synergism. Synergistic combinations ought to decrease the colony-forming unit concentration with a factor of at least 2 log_10_ per milliliter [49].

FIC index = MIC of antibiotic in combination/MIC of antibiotics alone.

FIC of the two drugs = FIC index of drug A + FIC index of drug B.

An FIC index of <0.5 indicates synergism, >0.5–1 indicates additive effects, >1 to <2 indicates indifference, and ≥2 is considered to be antagonism [49,50].

### 2.1. Combination of Beta-Lactams with Aminoglycosides and Fluoroquinolones

Antimicrobial synergy has conventionally been observed with combinations of beta-lactams and aminoglycosides for diseases of Gram-negative microorganism origin. The combinations of a beta-lactam and an aminoglycoside enables various mechanisms of action of destroying microorganisms [18,48,51,52,53]. Beta-lactam facilitated the disruption of Gram-negative bacilli’s cell walls and enabled aminoglycoside movement into the periplasmic space [54,55]. Though antibiotic synergy seems to be best proven for the combination of beta-lactam and aminoglycoside, similar information on synergistic effect has become apparent for “combinations of beta-lactams and aminoglycosides” [18,56,57,58].

#### 2.1.1. Combination Therapy of Beta-Lactams with Lipopeptide and Glycopeptide

Combination therapy with daptomycin and beta-lactams has also been studied in vitro; comparable to in vitro vancomycin studies, many combination-therapy studies have shown synergy, particularly among those with some degree of daptomycin resistance [59,60,61]. Gritsenko et al. [62] published a case series demonstrating clearance of refractory MRSA bacteremia in five patients treated with vancomycin and ceftaroline. Likewise, following microbiological failure or relapse with more conventional regimens (vancomycin monotherapy, daptomycin monotherapy, and daptomycin plus gentamicin), Dhand and Sakoulas, [63] reported a 2011 case series that evaluated seven patients treated with daptomycin and nafcillin or oxacillin. All patients were bacteremic for at least 5 days before receiving daptomycin in combination with an anti-staphylococcal beta-lactam, and all patients had documented bacterial clearance within 24–48 h of receiving daptomycin in combination with an anti-staphylococcal beta-lactam [63].

#### 2.1.2. Beta-Lactam/Beta-Lactamase Inhibitor Combinations

Several unique beta-lactam/beta-lactamase inhibitor combinations have recently been developed, which include ceftolozane/tazobactam, ceftazidime/avibactam, meropenem/vaborbactam, imipenem-cilastatin/relebactam, aztreonam/avibactam, cefepime/tazobactam, and ceftaroline/avibactam. The Food and Drug Administration has approved ceftolozane/tazobactam, ceftazidime/avibactam, meropenem/vaborbactam, and imipenem-cilastatin/relebactam for clinical use [64,65,66,67,68,69]. These new beta-lactam/beta-lactamase inhibitor combinations are effective against a wide range of bacteria, including the most common Gram-negative bacteria that cause complicated urinary tract infections and acute pyelonephritis. Furthermore, they have strong in vitro activity against a wide range of multidrug-resistant organisms [70,71,72,73].

#### 2.1.3. Clinical Cases of Combination Therapy

In medicine, combination therapy is frequently used when monotherapy does not produce an adequate therapeutic response. Patients at Seoul St. Mary’s Hospital who were under the age of 19 and had been diagnosed with *Pseudomonas aeruginosa* while having hematologic or oncologic comorbidities were studied in the retrospective observational study that was published by Kim et al. [74]. It was discovered that 36 different cases of P. aeruginosa infection, affecting 31 of the patients, had already occurred. The cohort’s *P. aeruginosa* infections were found to be moderately resistant to piperacillin-tazobactam and cefepime, with only 67.6% and 88.9% susceptibility, compared to these isolates being highly susceptible to amikacin, colistin, and ciprofloxacin (100%, 100%, and 97.2%, respectively). The cohort’s mean age was 9.5 ± 5.4. The “patients who did receive a combination therapy were treated with either piperacillin–tazobactam plus an aminoglycoside (16 (44.4%)), cefepime with an aminoglycoside (2 (5.6%)), or meropenem with an aminoglycoside (1 (2.8%)). The monotherapy treatments consisted of either meropenem (14 (38.9%)) or cefepime (3 (7.4%)”. Overall, it was discovered that monotherapies had a mortality rate of 17 (or 58.8%), whereas combination therapies had a mortality rate of 4 (or 21%). Cefepime or cefepime plus aminoglycoside were the most effective therapies, both of which had a 0% fatality rate.

Likewise, according to Ceravolo et al. [75] reports, a seven-year-old Caucasian child with neuronopathic Gaucher disease who was homozygous for L444P mutations was treated with a combination of substrate reduction therapy and enzyme replacement therapy. He had been receiving enzyme replacement therapy since he was 18 months old, and concurrent miglustat treatment started when he was 30 months old. Dosing was increased over the course of a month in accordance with his body surface area. After starting his miglustat therapy, he suffered mild diarrhea, which become progressively less frequent and severe. His hematological parameters and plasma angiotensin-converting enzyme activity returned to normal, and his splenomegaly was reduced. Additionally, there was a significant and consistent drop in plasma chitotriosidase. The patient displayed no symptoms of neurological impairment after receiving combination therapy for five years.

### 2.2. Nano-Materials Combinations with Antibacterial Drugs

Adjuvants are molecules that, when combined with drugs, make an ineffectual medicine efficient. They have hardly any antibacterial properties of their own [76], but extend the lifetime of antibiotics, and impede resistance mechanisms [77,78]. However, they habitually produce some harmful results due to drug-drug interactivities [36]. Nanomaterials are materials having a minimum of one of their dimensions not exceeding 100 nm, and they are the key constituents of nanotechnology [36,79]. Nanoparticles (NPs) act like magic bullets with the right concentration to target the delivery of the drug at the proper place and at a suitable time [80]. This is beneficial over conventional systems as they can surmount drug resistance given their multi-performance nature, as microorganisms will not be able to utilize multiple gene mutations at the same time [36].

Antibacterial agents can be combined with NPs to overcome antibiotic resistance and enhance their efficacy. They can reduce the dose and toxicity of antibiotics to be taken [81]. And since NPs act on bacteria via multiple targets and/or mechanisms, it is extremely difficult for bacteria to develop resistance. In other words, the likelihood of simultaneous mutations required for resistance formation is extremely low. This is especially unlikely when NPs are combined with antimicrobials [82]. As a result, the use of NPs in combination with antibiotics is regarded as a method for preventing the development of bacterial resistance [82]. Antibiotics used in conjunction with NPs are more effective against Gram-positive and Gram-negative bacteria, as well as drug-resistant bacteria. Aabed and Mohammed [83] demonstrated synergistic effects of AgNPs in combination with bacitracin, ciprofloxacin, tetracycline, and cefixime against *P. aeruginosa*, *E. coli*, *S. aureus*, and *Candida albicans*. In another study, ZnO NPs combined with Vancomycin, and ampicillin had a synergistic effect against MDR *Enterococcus feacium* [84].

## 3. Effect of Nanomaterials on Bacteria

Some distinguishing characteristics of nanomaterials that make them a potential preference for antibiotics are discussed below.

- They can simply infiltrate the cell membrane of bacteria and potentially harm its composition, which eventually causes cell lysis [85].
- The modality of action of nanomaterials’ antimicrobial effect is similar to the mechanism of antibiotics, such as cell membrane disruption, “reactive oxygen species (ROS)-facilitated oxidative stress, intracellular protein production inhibition, and leaking of intracellular components” [86].
- Several nanoparticles can serve as carriers for antibiotic drugs to distribute them efficiently to their action sites by reducing the drugs’ likely negative results [33].
- The retaining power of NPs in the body far exceeds that of antibiotics, and this could be beneficial for lasting therapeutic results [87].
- Nanomaterials can be highly functional with respect to their target and their goal because they may be efficient against the cells of bacteria without actually being noxious to animal cells [33].

### 3.1. Organic versus Inorganic Nanoparticles

Nanoparticles (NPs) may be classified as organic or inorganic based on the desirable action. Organic NPs consist of “liposomes, polymeric NPs, polymeric micelles, and solid lipid NPs (SLNs)” utilized in main treatments. Their main benefits comprise biodegradability, low systemic toxicity, compatibility, and handling of either hydrophilic or hydrophobic drugs. Nevertheless, organic NPs have some limitations, which include low encapsulation efficiency, short shelf life, poor stability at elevated temperatures, and a lack of tolerance for severe processing environments [88]. Inorganic NPs harbor exceptional physicochemical attributes due to their high surface volume, which makes them an extremely promising therapy as an antibacterial by overcoming the detriments of antibiotics and bulk metals [89]. For the synthesis of metal-based NPs, transition metals are likely to be the most suitable because they have partially filled d-orbitals that confer better redox activity on them, a characteristic that enables nanoparticle aggregation [90].

### 3.2. Types of Inorganic Nanoparticles

The most tested metallic NPs are copper, silver, gold, titanium, aluminum, zinc, and iron [91,92]. Compared to antibiotics, the different antibacterial mechanisms of NPs depend on their unusual crystal morphologies (edges and corners), size, large surface, and reactive sites [93]. They are reported to show wide-spectrum antibacterial properties against mycobacteria, Gram-positive and -negative organisms, and fungi. Nevertheless, their antibacterial properties differ amid the various kinds of nanoparticles as along with the diverse organisms [94,95].

### 3.3. Silver Nanoparticles 

Silver nanoparticles (AgNPs) have been reported as the most investigated nanomaterials for antimicrobial activity because of their broad range of actions against several microbes [96,97,98]. Silver compounds such as silver nitrate, metallic silver, and silver sulfadiazine are used for various medical uses including dental work, burns, disinfection of medical equipment wound treatment, and controlling bacterial contamination, among others [99]. Moreover, due to their multi-dimensional strategies to convey antimicrobial action, the possibility of resistance of bacteria to AgNPs is low [100].

**Figure 1:**
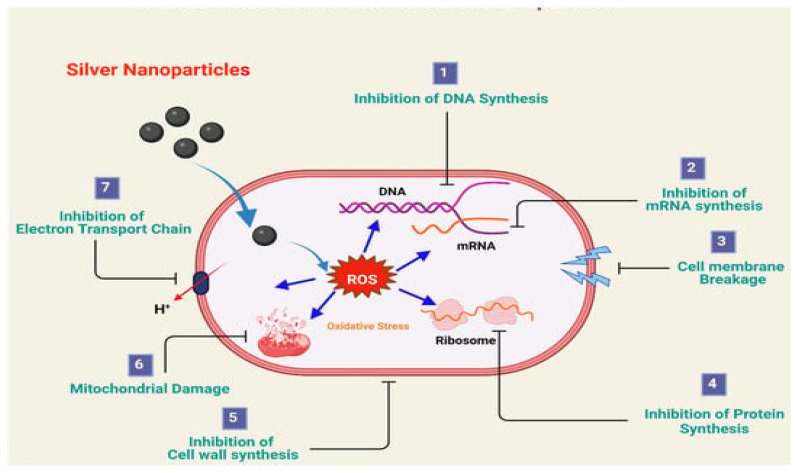
Antibacterial mechanism of silver nanoparticles. Source: Jain et al. [98] with permission to reuse the figure under a CC BY open access license.

**Figure 2:**
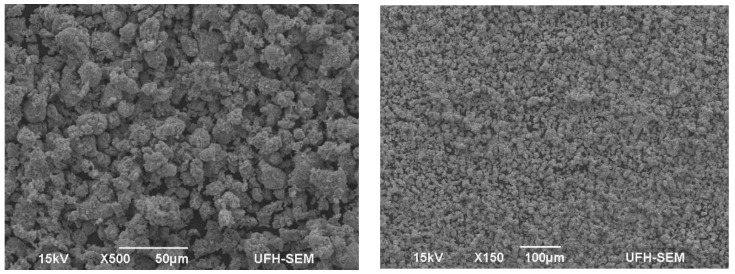
Scanning electron micrograph of silver nanoparticles (Adeniji et al. [84]).

Several researchers have proposed various antimicrobial actions for AgNPs, which include impairment of the external membrane of bacteria [101], interface with enzymes, and disintegration of the cellular elements [64], as shown in Figure 1 [98]. It was also reported that the antibacterial activity of AgNPs depends on their size and shape. In this respect, Lu et al. [102] affirmed that the antibacterial property and particle size of AgNPs do not correlate directly but inversely. The three distinct types of AgNPs with good antibacterial activity are spherical, rod-shaped, and truncated triangular AgNPs (Figure 2) [84,103]. The properties of these nanoparticles, which influence their activity towards bacterial pathogens, are presented in Table 1.

### 3.4. Zinc Oxide Nanoparticles (ZnO NPs)

**Figure 3:**
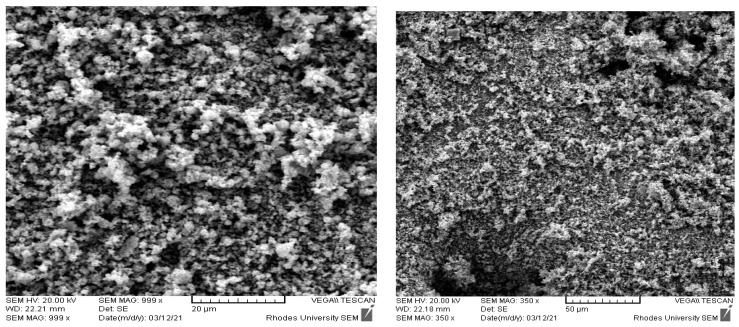
Scanning electron micrograph of Zinc oxide nanoparticles (Adeniji et al. [84]).

NPs of ZnO origin are well known to efficiently hamper the development of a broad range of susceptible and non-susceptible microorganisms, rising as optimistic candidates to combat antibacterial resistance [111,112]. Iron oxide (Fe_3_O_4_), copper oxide, and zinc oxide (ZnO) have antibacterial effects that enable their application in medical care [113]. “Regarding the intrinsic photocatalytic property of metal oxides, they generate ROS and become potent agents against bacteria [114,115]. Gelabert et al. [116] and Nagvenkar et al. [117] reported that the antibacterial mechanism of the NPs is linked partially to the dissolution of metal ions and the formation of ROS” (Figure 3). “ZnO releases Zn^2+^ in a liquid medium and is adsorbed at the surface of bacteria, where it interacts with functional groups in proteins and nucleic acids, obstructing enzyme activity and the normal physiological processes [118]. However, some authors demonstrated that Zn ions have little antimicrobial activity, implying that dissolution of Zn^2+^ might not be the main mechanism of action” [119,120].

Zinc oxide NPs can be produced through chemical and physical processes, such as chemical vapor deposition, solvothermal, sol-gel hydrothermal, laser exposer, and spray pyrolysis [121,122]. Zinc oxide has been found useful in photocatalysis, light-emitting diodes, UV filtration, solar cells, piezo-electric transducers, memory devices, and photodetectors [121,123]. ZnO is also used “in the structure of electrochemical sensors and biosensors” [124,125], the food industry [126], wastewater treatment [127], sunscreens [128], composites [129], dental cement, drug delivery system, and in cancer treatment [130]. Thus, Zinc oxide NPs might be utilized as prospective antimicrobials against several pathogenic organisms [131].

### 3.5. Other Nanoparticles

**Figure 4:**
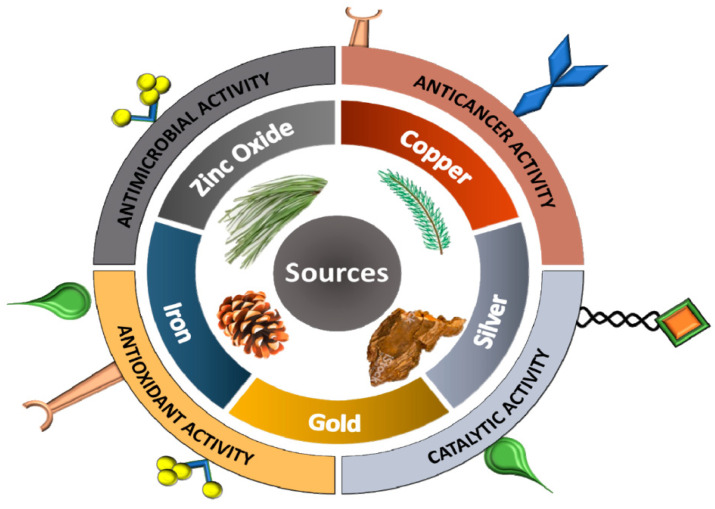
Metal-derived NPs and their application. Source: Bhardwaj et al. [139] with permission to reuse the figure under a CC BY open access license.

Several other NPs have been documented, including Si, SiO_2_ [132], MgO [133], CaO [134], Al_2_O_3_ [135], and bismuth [136]. Yamamoto et al. [137], “reported that the generation of superoxide on their surface was the major reaction mechanism of antibacterial action by CaO and MgO”. It was also reported that the “antibacterial mechanism of Al_2_O_3_ is dependent on the interaction between NPs and bacterial cell membranes” [138]. Metal-derived NPs and their application are shown in Figure 4 [139].

## 4. Mode of Action of Nanomaterials

**Figure 5:**
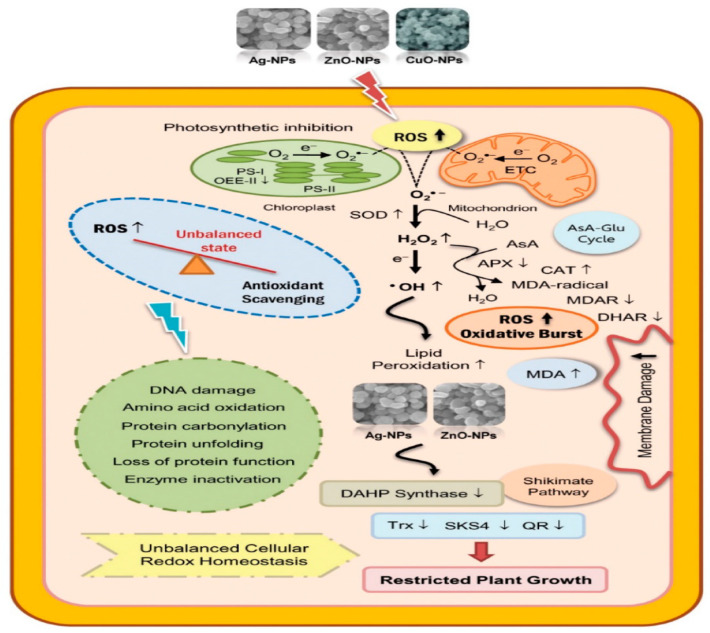
Mode of action of nanoparticles. Adapted from Hossain et al. [131] with permission to reuse the figure under a CC BY open access license.

NPs have an antimicrobial property that can subjugate common resistant mechanisms such as reduced “cell permeability, alteration of target sites, enzyme inactivation, and increased efflux through overexpression of efflux pumps to scarper from the antibacterial effect of antibiotics” [139]. The antimicrobial action of nanoparticles against MDR organisms and biofilms is based on several features: their high surface area in connection with bacteria via electrostatic attraction, hydrophobic interactions, or van der Waals forces on the NPs size and stability; and how concentrated the drug is [140,141]. The interplay of nanoparticles with bacteria usually activates oxidative stress actions, inhibition of the enzyme, deactivation of protein, and changes in gene expression Typical mechanisms of antibacterial action are associated with metal ion release, oxidative stress, and non-oxidative actions, as shown in Figure 5 [85,142].

Due to electrostatic interactions, there is an attraction between the bacteria cell walls, which have negative charges, and the surfaces of the NPs, which are positively charged. Alternately, metal-based NPs with positive charges build a powerful bond with membranes following the intrusion of cell walls and, subsequently, enhance their permeability [143]. Moreso, NPs are also able to discharge metal ions from the extracellular space, which are proficient in penetrating the cell and disturbing genetic activities [143]. Metal ions of NPs inside the cell can induce the making of ROS. “Oxidative stress instigated by ROS is among the utmost critical mechanisms aiding the antimicrobial effect of nanoparticles [144,145]. ROS are raw by-products of cellular oxidative metabolism with substantial essential functions in the modulation of cell survival and death, cell signaling, and differentiation” [146] The oxidative stress produced leads to glutathione oxidation, hence defeating the antioxidant protective mechanism of the microorganisms against ROS. The metal ions could then move freely within the cellular structure disrupting cell functions [143].

Membrane infiltration can also be accomplished by “interactions with surface lipids [147]. The curative application of NPs is improved by their capability to bestow physical protection on the mechanisms of microbial resistance [148]. Ansari et al. [138] documented that the buildup of NPs in the bacterial cell wall results in an irregularly shaped pit, and perforation and interferes with metabolic activities. Research carried out by Joost et al. [149] reported that therapy with TiO_2_ NPs amplified the bacterial cell volume, bringing about membrane leakage. Furthermore, NPs bonded with antibiotics exhibit co-adjuvant “effects against bacteria, interdict biofilm formation, and are employed to fight MDROs [87,120]. “While inadequate membrane transport confines the efficacy of several drugs [150], drug-loaded NPs’ vehicles can move into host cells using endocytosis, enabling their intracellular entry” [85].

### 4.1. Advantages of Nanomaterials in Combating MDR Pathogens

Nanomaterials might be efficaciously modified to have an antibacterial property with no noxious side effects based on their unique physicochemical properties [87]. In the same vein, they can be distributed in appropriate and affordable means with reduced administration frequency by numerous paths [151]. “Synergistic antibacterial activity, improved solubility, and suspension of drugs are additional advantages of nanomaterials. Besides their excellent antibacterial properties, nanomaterials can be used as carriers for the delivery of antimicrobial moieties to regions of poor absorption in the body” [33]. The benefits of nanomaterials as antibacterial drug delivery vehicles are listed below.

- As a result of the inadequate membrane transport process of some drugs, their impact on intracellular disease-causing organisms is constricted. Meanwhile, the controllable size of the NPs aids in designing targeted antibiotics [143,152].
- The time of drug retention in the blood can be enhanced as a result of using NPs as an antimicrobial drug delivery vehicle [153].
- The solubility of nanomaterials in the bloodstream is allowed by the surface chemistry of NPs [33].
- Opsonization is an additional biological impediment where the physicochemical properties of nanomaterials have been well applied for the efficient delivery of antibacterial drugs to the site of action. It also allows a high measure at the site of infection site and therefore reduces the harmful effects [154,155]. For example, vancomycin is an effective Gram-positive bacteria drug but can be harmful to the kidney and ear [85]. In that regard, “Qi et al. [156] documented that the vancomycin-modified mesoporous silica NPs can be aimed at a particular Gram-positive disease-causing microorganism and selectively exterminate them over macrophage-like cells” [130].
- Antibiotics can be protected from damage to the chemical reaction and resistance against targeted bacteria by nanomaterials. It has been proven by many researchers that several NPs can “overcome the traditional efflux mechanism of bacteria cells that often obstruct the uptake of antibiotics by the cells” [157]. For example, “Liu et al. [158] reported that the dendrimers could impede P-glycoprotein-mediated efflux in the gastrointestinal tract”.

### 4.2. Antibacterial Effect of Nanomaterials-Antibiotics Combination

Functionalization of NPs with antibiotics has the potential to fight against resistant bacteria because NPs can take antimicrobial agents to the sites of infection and lessen the toxicity and dosage of drugs [155]. Combining nanomaterials with antibiotics has been widely reported as being potent in combating bacterial resistance [127]. Panacek et al. [159] and Scandorieiro et al. [160] documented the synergistic antimicrobial efficacy of AgNPs and antibiotics against *Staphylococcus aureus*, beta-lactamase- or carbapenemase-producing *Escherichia coli*, *Pseudomonas*. *Aeruginosa,* and *Acinetobacter baumannii* strains at very small concentrations via the infiltration of the bacterial cell membrane and the intrusion with essential molecular pathways, creating distinctive antimicrobial mechanisms [161].

To ascertain the interactive impact of antibiotics and nanoparticles, the fold increment in the diameter of the inhibition zone of each antibiotic after combination with nanoparticles is determined according to Sindhu et al. [162].

*The fold increase* = (*b*^2^ − *a*^2^)/*a*^2^

“where; (*a*) is the inhibition zone of antibiotic alone and (*b*) is the inhibition zone of antibiotic plus nanoparticles”.

The nanomaterial–antibiotics blend has efficaciously mired the growth of MDR microorganisms according to various reports. Silver nanoparticles with ciprofloxacin [161], vancomycin [163], and clotrimazole [164] have effectively prevented the growth of Vancomycin-resistant *Enterococci* (VRE) and Methicillin-resistant *Staphylococcus aureus* (MRSA) species. Gold NPs with vancomycin [165] or ampicillin [166], and ZnO NPs along with ciprofloxacin [167], similarly displayed antibacterial effects against MRSA and MDR *A. baumannii,* respectively. The efficiency of drugs combined with nanoparticles was the same in Gram-positive and -negative microorganisms, in contrast to the complexity of exterminating MDROs with antibiotics only [155]. Thus, further studies are needed on the blend of nanomaterials with antibiotics, as this could offer an unimaginable breakthrough in the treatment of diseases caused by pathogenic MDR organisms.

## 5. Cytotoxicity of Nanomaterials

The concerns over the use of NPs are local and systemic noxious problems, plus harmful effects on helpful bacteria in humans [87,168]. Kandi and Kandi [169] reported that nanoparticles and their toxic disintegration products could bring about hemolysis and interfere with blood clotting pathways. Meanwhile, the mechanism of toxic problems remains unclear, but noticeably, large-sized nanoparticles have a higher tendency of endangering human health [170].

AgNPs are reported as the most powerful nano weapon in the fight against bacterial diseases [103,171]. However, resistance to AgNPs is now increasingly reported because of the genetic modification of bacteria [172]. The deposition of silver nanoparticles in the liver, lungs, spleen and other organs is responsible for the potential harm and dysfunction of such body organs and the extreme reduction of their effectiveness [155]. The oxidative harm of CuO NPs and damage to DNA caused by zinc oxide nanoparticles or TiO_2_ NPs have limited their usage [155]. The buildup of metallic NPs in the tissues can lead to long-term toxicity, like hepatotoxicity, or nephrotoxicity, among others [173,174,175]. Nevertheless, some in vivo investigations have indicated there are no obvious life-threatening poisonous effects connected to nanoparticles [174,175,176]. Reports about the biocompatibility of various nanomaterials in cases wherein toxicity depends on the size, concentration, and time of treatment are numerous [177]. For example, Naskar et al. [178] stated that the Ag–ZnO–graphene nanocomposite is not lethal at low amounts but poisonous when the levels are higher. Nevertheless, nanoparticles have become known as the alternative antibacterial strategy for the fight against biofilms and the treatment of serious bacterial infectious diseases [179].

### Approaches to Addressing Nanomaterials Toxicity Dilemma

Nanoparticles have long been regarded as a potential solution to the rising resistance to common antibiotics and the emergence of multidrug-resistant bacteria. Despite the enormous potency of these nanoparticles, reports on bacterial resistance to nanoparticles are starting to emerge. Its prevalent clinical application raises the prospect of resistance to these potential biomolecules [180,181]. Some studies have shown that AgNPs have cytotoxic effects in various cell lines based on size, shape, concentration, or capping agent [182,183,184]. As a result, combinations with other antimicrobial treatments have been suggested to enhance antimicrobial properties while decreasing AgNP cytotoxicity. Many devices have already been employed to combat nanomaterial toxicity. The best tactic is to cap the nanoparticle with a biocompatible polymer such as *polyethylene glycol* (PEG) or chitosan. PEG is a synthetic polymer that is well-suited for biomedical applications such as bioconjugation, drug delivery, and bio-sensing [185]. Reports have shown that PEG capping NPs reduces the noxiousness of NPs and enhances biological compatibility [177,186]. Likewise, these polymers have their own antibacterial properties. Hence, the capping of nanoparticles with *polyethylene glycol* or chitosan not only improves their biological compatibility but the capping materials also exterminate bacterial cells collectively with nanoparticles [186,187] Abdalla et al. [188], for example, tested polyvinyl alcohol (PV) and chitosan (C) as capping agents in AgNPs against clinical isolates of *Staphylococcus epidermis*, *Staphylococcus aureus*, *Klebsiella. pneumoniae*, and *Escherichia coli*. Surprisingly, this combination had a beneficial antibacterial effect and inhibited biofilm formation in all isolates tested. Chia et al. [189] also described the use of a silica coating to reduce the toxicity of ZnO NPs. Doping is also an efficient approach to lessen the perniciousness of nanomaterials. In this regard, Xia et al. [190] stated that Fe-doped ZnO lessened toxicity in rodent lungs and zebrafish embryos by reducing the dissolution of zinc oxide nanoparticles. In addition, Limayem et al. [191] investigated the antibacterial activity of chitosan NPs, ZnO alone, and a combination of chitosan and ZnO against MDR and wild-type strains. On MDR *E. coli* ZnO combined with chitosan demonstrated a synergistic effect as well as with MDR *Enterococcus faecium*. Later, Mehta et al. [192] tested biofilm activity against MDR *Enterococcus faecium* in a lipid micelle with the previously described composite (ZnO-chitosan composite). Surprisingly, the results showed a 50% reduction in bacterial biofilm size when compared to chitosan and ZnO alone. Still, it should be stated that a well-organized review is required before any clinical application of nanomaterials as drugs for antimicrobial activity.

## 6. Limitations and Strengths of the Application of Nanoparticles

Although NPs have the potential to treat infectious diseases, several challenges remain for their clinically effective translation, including further evaluation of their interactions with cells, tissues, and organs; optimal dose; identification of appropriate administration routes; and toxicity upon acute and long-term exposure [120,193]. It is critical to consider nanoparticle dosage. Drugs that are beneficial at low doses (concentration) may be toxic at high doses. Most studies report varying concentrations, and the number of cells exposed is rarely reported. Because nanoparticles have such promising potential, one important goal of the nanomaterials research community is to synthesize nanoparticles or nanoparticles that can conjugate very effectively at low doses (concentration). Non-toxic biological materials that can increase the potency of nanoparticles without increasing the concentration that may be toxic to biological systems should be studied. The combination of different nanoparticles can also aid in dosage reduction. Individual nanoparticles are also less effective than nanocomposites. As a result, more emphasis should be placed on their formulation [194]. Furthermore, creating nanoparticles that can bind to proteins, polysaccharides, or small bioactive compounds could be important in enhancing their antibacterial properties. The combination of nanoparticles and antibiotics significantly reduces the number of antibiotics that must be administered. This helps to reduce the toxicity of several antibiotics as well as the acquisition of resistance. Combination therapy will set the stage for nanoparticles to be used as adjuncts to the existing antibiotics, assisting in the reduction of resistance associated with the majority of bacteria.

## 7. Conclusions and Recommendations

MDR pathogens are becoming an emergent public health crisis, making many healthcare-related diseases demanding to treat with existing antibiotics. The use of nanomaterials provides a possible approach to controlling diseases caused by these pathogens. Nanoparticles demonstrating antibacterial properties can target numerous biomolecules and potentially lessen or eradicate the evolution of MDR. At the same time, the transformation of NPs to medical use necessitates suitable techniques for the preparation of NPs and comprehensive knowledge of the physicochemical distinctive features, in silico and in vivo effects, biodistribution pharmacokinetics, and pharmacodynamics of nanoparticles. Precise blends of NPs and antibiotics can help inhibit the occurrence of resistance or drive resistant bacteria back toward drug sensitivity. Furthermore, the clinical data on the nanomaterial-based antibacterial property are scanty, so there is a need for more in-depth studies, including in vivo findings towards a successful translation of nanomaterials to medical applications in attacking MDR organisms.

## Figures and Tables

**Table 1:** Properties of nanoparticles that influence their activity toward bacterial pathogens.

Property	Functions	References
Particle Size	“Smaller Ag-NPs have larger specific surface areas, which result in higher antimicrobial activity”. Because there is a higher chance of being in contact with, and transient on, the cellular membrane of the organism than with bigger nanoparticles.	[64,104,105]
Particle Shape	Ag-NPs with various forms can result in various microbial cell injury degrees through interacting with periplasmic enzymes. For example, cube-shaped silver nanoparticles demonstrate more potent antimicrobial effect than sphere-shaped and wire-shaped silver nanoparticles with comparable diameters, implying that the shape impact on antimicrobial activities is because of the surface area and facet reactivity.	[105,106,107]
Roughness	“As the roughness of Ag-NPs increases, the size and the surface area-to-mass ratio promotes the adsorption of bacterial proteins, which is followed by a reduction in bacterial adhesion”.	[64]
Environmental Condition	Temperature and pH cause considerable differences in antimicrobial effect. For example, the temperature of the environment has a strong impact on antimicrobial activity due to its impact on ROS generation rate. Likewise, “at low pH, the surfaces of the NPs were positively charged, which is beneficial to the interaction with the negatively charged groups of the bacterial cell barrier, inducing strong multivalent electrostatic regulation”.	[64,108]
Doping Modification	Prevents the assemblage of gaps and permits their dispersion in aqueous conditions or other hydrophilic media.	[109]
Zeta Potential	AgNPs with a positive surface charge are susceptible to the fact that they are absorbed on bacterial surfaces, in contrast to their counterparts with negative charge.	[110]

## Data Availability

Not applicable.
